# COVID-19 dynamic modeling of immune variability and multistage vaccination strategies: A case study in Malaysia

**DOI:** 10.1016/j.idm.2024.12.011

**Published:** 2024-12-19

**Authors:** Emmanuel A. Nwaibeh, Majid K.M. Ali

**Affiliations:** a School of Mathematical Sciences, Universiti Sains Malaysia, 11700, Glugor, Pulau Penang, Malaysia; b Nigeria Maritime University Okerenkoko, 332105, Warri, Delta, Nigeria

**Keywords:** COVID-19, Hybrid-immune and immunodeficient, Vaccination, Hopf bifurcation, Parameter estimation, Simulations

## Abstract

Hybrid-immune and immunodeficient individuals have been identified by the World Health Organization as two vulnerable groups in the context of COVID-19, but their distinct characteristics remain underexplored. To address this gap, we developed an extended *SIVS* compartmental model that simulates the spread of COVID-19 and the impact of administering three doses of the vaccine (first, second, and booster). This study aims to provide insights into how these vulnerable populations respond to vaccination and the dynamics of waning immunity. Using real-time data from the Ministry of Health of Malaysia (May 2023–April 2024), we estimated key parameters through numerical methods and fitted the model to the data using MATLAB's lsqcurvefit package. We carried out stability and equilibrium analyses, computed the basic reproduction number (*R*_0_), and identified conditions for Hopf bifurcation. Sensitivity analysis highlights the parameters with the greatest impact on infection dynamics. The calculated basic reproduction number and stability results suggest that with current vaccination rates, COVID-19 will persist in the population over an extended period. Our findings provide valuable information for public health agencies, offering recommendations for vaccination strategies targeting hybrid-immune and immunodeficient groups. These insights can inform decisions about vaccine booster schedules and resource allocation to better manage the pandemic.

## 1 Introduction

The global COVID-19 pandemic has spurred the development of various mathematical models aimed at predicting and controlling the virus's spread. As new variants emerge and second waves occur, compartmental models have been widely used to study the dynamics of infectious diseases, including COVID-19, as demonstrated in works by researchers such as (Anand & Mayya, 2020; Anfinrud, Stadnytskyi, Bax, & Bax, 2020; Jayaweera, Perera, Gunawardana, & Manatunge, 2020; Kwon et al., 2021; Lai et al., 2021).

Compartmental models have a long history, originating from the works of (Kendall, 1956; Kermack & McKendrick, 1927; Ross, 1916; Ross & Hudson, 1917), and have since been applied to a variety of diseases including Ebola (Dé et al., 2019), malaria (Eikenberry & Gumel, 2018), and HIV. These models have also been extended to non-biological applications such as computer worms, viruses (Nwaibeh, Kamara, Oladejo, & Adamu, 2019), and even scam rumors (Nwaibeh & Chikwendu, 2023).

Recent advancements in COVID-19 modeling have introduced new parameters and adjustments to enhance prediction accuracy (Gonzalez-Parra, Mahmud, & Kadelka, 2024; Okada & Nishiura, 2024). One key area of focus is the immune status of individuals, which plays a critical role in determining the spread of the virus. While studies have incorporated non-pharmaceutical interventions and immune status (Althobaity, Wu, & Tildesley, 2022), the distinction between individuals with strong immune responses (hybrid-immune) and those with weaker immune systems (immunodeficient) has not been thoroughly explored using deterministic modeling approaches. For instance (Manaqib, Mahmudi, & Prayoga, 2023), modeled two susceptible classes based on compliance with preventive guidelines, but no model has yet focused on susceptibility based on immune strength.

To address this gap, we developed a new *SIV*_1_*V*_2_*V*_*b*_*S* model that accounts for varying levels of immunity (strong and weak) and incorporates the three recommended doses of the COVID-19 vaccin e (first dose, second dose, and booster). This model uses daily COVID-19 reports from the Ministry of Health Malaysia to analyze the spread of the virus and assess the long-term impact of the vaccination program. Additionally, the model accounts for the waning immunity of the booster dose, which adds to its realism and utility in policy planning.

The main objectives of this study are to analyze the dynamics of COVID-19 spread among populations with heterogeneous susceptibility (hybrid-immune and immunodeficient individuals), to evaluate the effectiveness and long-term impact of the three-dose vaccination strategy in Malaysia, and to estimate key epidemiological parameters while providing insights for enhancing intervention and control strategies.

In conclusion, the novelty of this work lies in its focus on the combination of heterogeneous susceptibility, the complete three-dose vaccination program, and the waning period of the booster dose. As of this study, no similar research has been conducted in Malaysia, making this a crucial contribution to understanding the COVID-19 pandemic's dynamics and informing future public health interventions.

## 2 Model formulation

In order to describe the post-COVID-19 dynamics of the administration of first, second and booster shots of vaccinations as prescribed by the World Health Organization in a heterogeneous population, using the Malaysian data as a case study and to account for the dynamics of hybrid-immune and immunodeficient individuals, a new *SIV*_1_*V*_2_*V*_*b*_*S* model was proposed. The model comprised three main classes: the susceptible individuals, infected individuals and the vaccinated individuals. The motivation behind this model or work is the fact that a number of works have proven that some people have strong immune systems that provide better protection against the virus, while others have weaker immune systems and are more vulnerable to COVID-19 virus (Bienvenu, Noonan, Wang, & Peter, 2020; Nizami & Mujeebuddin, 2020; Paul, Steptoe, & Fancourt, 2021; Widjaja et al., 2021), thereby grouping the set of susceptible persons into two classes; also, as of the time of this work, no work has been carried out in Malaysia that present the dynamical impact of the administration for the first, second and booster shot of the COVID-19 vaccine using the recent daily data report. The main objective of this work is formulate a simplified general model that would appropriately and accurately describe the outlined factors. Hence, the following assumptions are made.

1. The susceptible population is divided into two distinct groups, hybrid-immune (*S*_*h*_) and immunodeficient (*S*_*c*_), based on evidence of varying immune responses (Bienvenu et al., 2020; Nizami & Mujeebuddin, 2020; Paul et al., 2021; Widjaja et al., 2021). All infectious individuals, whether symptomatic or asymptomatic, are included in the infected compartment (*I*).
2. Based on studies that show COVID-19 can spread through direct contact or airborne particles without a defined incubation period (Anand & Mayya, 2020; Anfinrud et al., 2020; Jayaweera et al., 2020; Kwon et al., 2021; Lai et al., 2021), we classify all exposed individuals as infectious immediately. We employ the Density-Dependent Incidence Rate (*βSI*), which assumes a constant contact rate.
3. Research shows that COVID-19 vaccine efficacy wanes after a period of time (Khan & Tanimoto, 2024; Menni et al., 2022), so we assume that individuals progress through the first dose, second dose, and booster classes before vaccine immunity wanes. To reflect the waning immunity, we introduce a time delay in the booster class, allowing individuals to return to the susceptible class.
4. The model includes a recruitment rate (*π*) for the susceptible class, a natural death rate (*μ*) for all compartments, and a virus-induced death rate (*δ*) for the infected class.

**Fig. 1:**
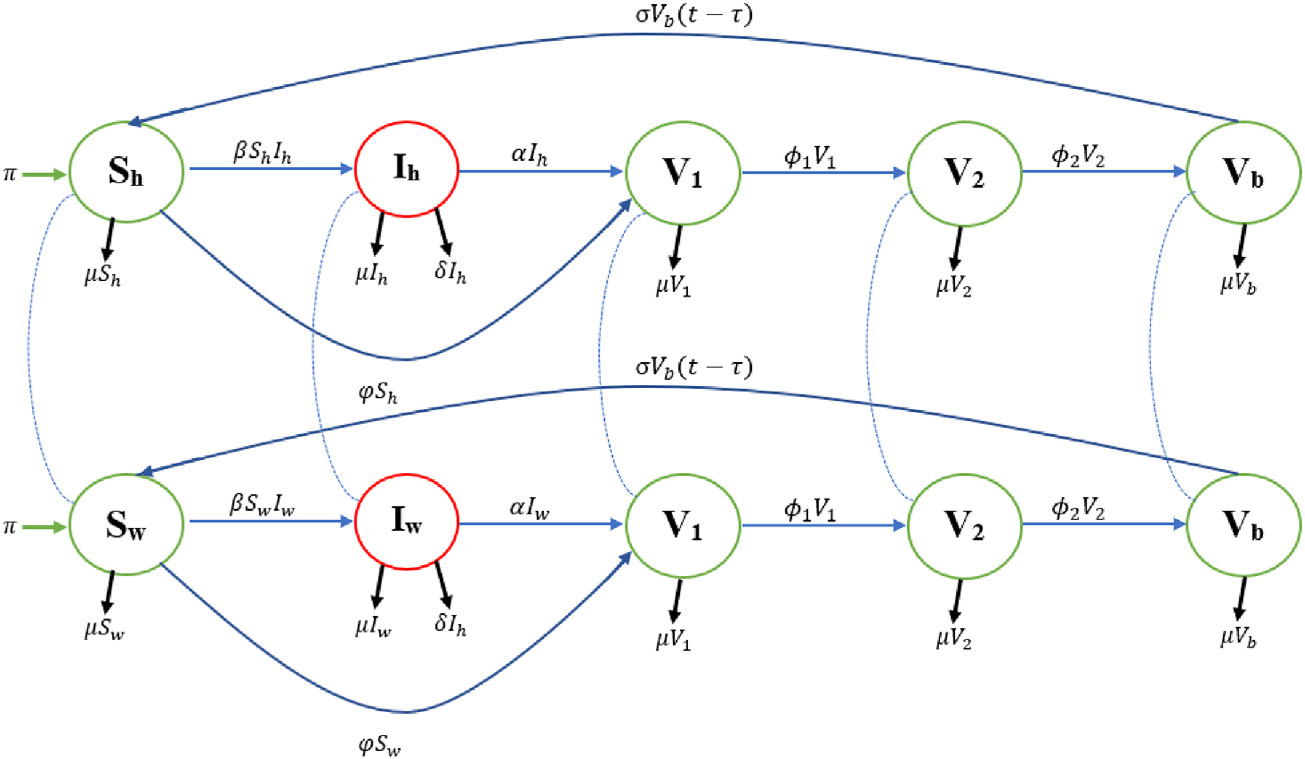
Epidemiological interaction dynamics of the COVID-19 *S*_*h*_*I*_*h*_*V*_1_*V*_2_*V*_*b*_*S*_*h*_ and *S*_*w*_*I*_*w*_*V*_1_*V*_2_*V*_*b*_*S*_*w*_ model.

Since we have two distinct models that are the same, it is enough to analyze one, thus, let *S* = *S*_*h*_ = *S*_*c*_, *I* = *I*_*h*_ = *I*_*c*_ while can we distinctly analyze the two in the numerical simulations to obtain the estimated value of each influencing parameter. Based on our assumptions and the flow of transmission of COVID-19 within groups of people as depicted in Fig. 1, we have the following system of differential equations:

(1) $$\begin{array}{ll} \frac{dS}{dt} & =\pi +\sigma V_{b}\left(t-\tau \right)-\beta SI-\mu S\\ \frac{dI}{dt} & =\beta SI-\left(\alpha +\delta +\mu \right)I\\ \frac{dV_{1}}{dt} & =\varphi S+\alpha I-\left(\phi_{1}+\mu \right)V_{1}\\ \frac{dV_{2}}{dt} & =\phi_{1}V_{1}-\left(\phi_{2}+\mu \right)V_{2}\\ \frac{dV_{b}}{dt} & =\phi_{2}V_{2}-\sigma V_{b}\left(t-\tau \right)-\mu V_{b} \end{array}$$

where the description of the state variable of the model are presented in Table 1, while the description of the parameters of the model is presented in Table 2. Fig. 1 shows the flow chart of the two distinct model *S*_*h*_*I*_*h*_*V*_1_*V*_2_*V*_*b*_*S*_*h*_ and *S*_*c*_*I*_*c*_*V*_1_*V*_2_*V*_*b*_*S*_*c*_ model.

Lemma 2.0.1 *IfS*(0) ≥ 0, *I*(0) ≥ 0, *V*_1_(0) ≥ 0, *V*_2_(0) ≥ 0, *V*_*b*_(0) ≥ 0, *then all the solution of the system (1) in* $R_{+}^{5}$ *are non-negative and bounded whenever* *t* > 0.

**Fig. 2:**
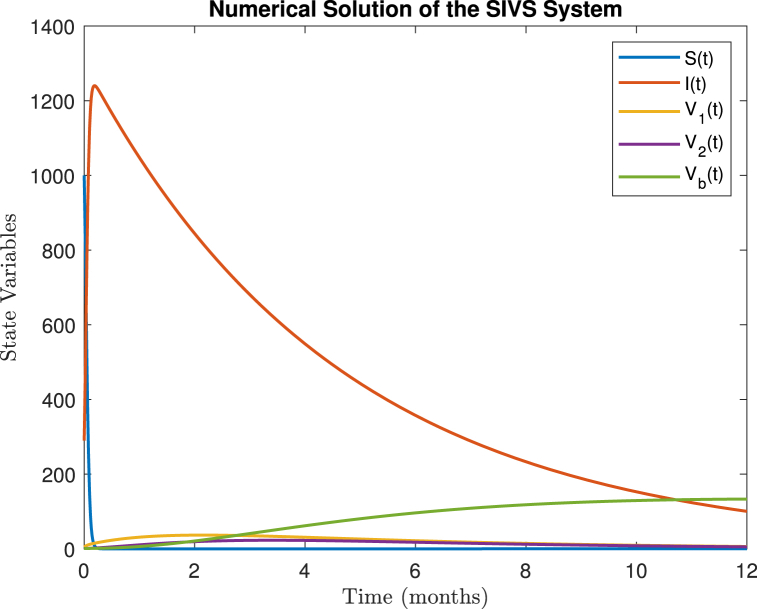
Numerical solution of the *SIV*_1_*V*_2_*V*_*b*_*S* model.

**Table 1:** Description of the state variables of the model.

Variables	Description
*S*_*h*_	Hybrid-immune susceptible group/individuals at a given time (*t*)
*S*_*c*_	Immune-compromised/deficient susceptible group/individuals at a given time (*t*)
*I*_*s*_	Group of hybrid-immune individuals who are infectious with COVID-19 at a given time (*t*)
*I*_*a*_	Group of immune-deficient individuals who are infectious with COVID-19 at a given time (*t*)
*V*_1_	Group of persons who have received the first dose of COVID-19 vaccination
*V*_2_	Group of persons who have received the second dose of COVID-19 vaccination
*V*_*b*_	Group of persons who have received the booster dose of COVID-19 vaccine

**Table 2:** Parameter description.

Parameter	Parameter Description
*π*	The of new cases into the susceptible class
*σ*	The rate at which booster dose vaccinated individuals become susceptible after losing immunity.
*β*	The effective contact rate.
*μ*	Natural birth rate and death.
*α*	The rate at which the infectious class are being vaccinated.
*δ*	Death as a result of COVID-19 virus.
*φ*	The rate at which the susceptible are being vaccinated.
*ϕ*_1_	The rate at which the first shot vaccinated receives the second shot.
*ϕ*_2_	The rate at which the second shot vaccinated receives the booster dose.
*τ*_1_	Vaccine immunity waning delay period.

*Proof*. Since all the dependent variables are that of human population they are non-negative, thus it is assumed that the parameters are also non-negative. Now, let $\left(S^{*},I^{*},V_{1}^{*},V_{2}^{*},V_{b}^{*}\right)$ be the solution of system (1), in particular we want to show that *S*∗(*t*) ≥ 0 for *t* ≥ 0. Suppose by contradiction that *S*∗(*t*) is not always non-negative for *t* ≥ 0 and let *t*_1_ be the first time that *S*∗(*t*) = 0, then *S*′(*t*_1_) < 0. But from the given system (1) *S*′(*t*_1_) = *π* + *σV*_*b*_*e*^−*λτ*^ − *βSI* − *μS*, since the state variables are non-negative, in particular taking *I* = *V*_1_ = 0,we obtain *S*′(*t*_1_) = *π* ≥ 0 which is a contradiction. Therefore, *S*∗(*t*) ≥ 0 whenever *t* ≥ 0. Following the same step we can show that *I*∗(*t*) ≥ 0, $V_{1}^{*}\left(t\right)\geq 0$, $V_{2}^{*}\left(t\right)\geq 0$, $V_{b}^{*}\left(t\right)\geq 0$ whenever *t* ≥ 0. Hence, the solution of system (1) is non-negative. For the boundedness of the system, observe that the sum of the compartment at any given time *t*, say $N^{\prime }\left(t\right)=\pi -\mu \left(S\left(t\right)+I\left(t\right)+V_{1}\left(t\right)+V_{2}\left(t\right)+V_{b}\left(t\right)\right)=\pi -\mu N\Rightarrow N\left(t\right)\leq \frac{\pi }{\mu }-\frac{1}{\mu }\Rightarrow dN\leq \frac{\pi }{\mu }$, thus the solution is bounded.

## 3 Equilibrium, stability analysis and existence of Hopf bifurcation

In this section, the equilibrium points and the stability of system (1) is presented.

### 3.1 Equilibrium points

The model of for system (1) has the following equilibria: the disease-free or COVID-19-free equilibrium point $\xi_{1}=\left(S^{*},I^{*},V_{1}^{*},V_{2}^{*},V_{b}^{*}\right)$ and the endemic point equilibrium point $\xi_{2}=\left(S^{*},I^{*},V_{1}^{*},V_{2}^{*},V_{b}^{*}\right)$ where,

(2) $$\xi_{1}^{*}=\left(\begin{array}{ll} S^{*} & =\frac{\pi \left(\mu^{3}+\mu^{2}\sigma +\mu^{2}\phi_{1}+\mu^{2}\phi_{2}+\mu \sigma \phi_{1}+\mu \sigma \phi_{2}+\mu \phi_{1}\phi_{2}+\sigma \phi_{1}\phi_{2}\right)}{\mu^{4}+\mu^{3}\sigma +\mu^{3}\phi_{1}+\mu^{3}\phi_{2}+\mu^{2}\sigma \phi_{1}+\mu^{2}\sigma \phi_{2}+\mu^{2}\phi_{1}\phi_{2}+\mu \sigma \phi_{1}\phi_{2}-\varphi \phi_{1}\phi_{2}\sigma },\\ I^{*} & =0,\\ V_{1}^{*} & =\frac{\pi \varphi \left(\mu^{2}+\mu \sigma +\mu \phi_{2}+\sigma \phi_{2}\right)}{\mu^{4}+\mu^{3}\sigma +\mu^{3}\phi_{1}+\mu^{3}\phi_{2}+\mu^{2}\sigma \phi_{1}+\mu^{2}\sigma \phi_{2}+\mu^{2}\phi_{1}\phi_{2}+\mu \sigma \phi_{1}\phi_{2}-\varphi \phi_{1}\phi_{2}\sigma },\\ V_{2}^{*} & =\frac{\left(\sigma +\mu \right)\pi \phi_{1}\varphi }{\mu^{4}+\mu^{3}\sigma +\mu^{3}\phi_{1}+\mu^{3}\phi_{2}+\mu^{2}\sigma \phi_{1}+\mu^{2}\sigma \phi_{2}+\mu^{2}\phi_{1}\phi_{2}+\mu \sigma \phi_{1}\phi_{2}-\varphi \phi_{1}\phi_{2}\sigma },\\ V_{b}^{*} & =\frac{\pi \phi_{1}\phi_{2}\varphi }{\mu^{4}+\mu^{3}\sigma +\mu^{3}\phi_{1}+\mu^{3}\phi_{2}+\mu^{2}\sigma \phi_{1}+\mu^{2}\sigma \phi_{2}+\mu^{2}\phi_{1}\phi_{2}+\mu \sigma \phi_{1}\phi_{2}-\varphi \phi_{1}\phi_{2}\sigma } \end{array}\right)$$

Observe immediately that $\xi_{1}^{*}\geq 0\;if\;only\;\mu \left(\phi_{1}+\mu \right)\left(\phi_{2}+\mu \right)\left(\sigma +\mu \right)\geq \sigma \varphi \phi_{1}\phi_{2}$.

(3) ξ1∗=S∗=α+δ+μβ,I∗=−μ5+α+δ+σ+ϕ1+ϕ2μ4+α+δ+σ+ϕ2ϕ1+α+δ+σϕ2+α+δσ−βpiμ3+α+δ+σϕ2+α+δσ−βpiϕ1+α+δσ−βpiϕ2−βpiσμ2+α+δ−φσ−βpiϕ2−βpiσϕ1−piβσϕ2μ−σϕ1ϕ2αφ+βpi+δφμ4+α+δ+σ+ϕ1+ϕ2μ3+α+δ+σ+ϕ2ϕ1+α+δ+σϕ2+α+δσμ2+α+δ+σϕ2+α+δσϕ1+σϕ2α+δμ+δσϕ1ϕ2β,V1∗=−α−φμ2+α−2φα+δμ−α2φ+−βpi−2δφα−δ2φμ+ϕ2μ+σμ4+α+δ+σ+ϕ1+ϕ2μ3+σ+ϕ1+ϕ2α+δ+σ+ϕ1ϕ2+δ+ϕ1σ+δϕ1μ2+σ+ϕ1ϕ2+σϕ1α+δ+ϕ1σ+δϕ1ϕ2+σδϕ1μ+δσϕ1ϕ2β,V2∗=−α−φμ2+α−2φα+δμ−α2φ+−βpi−2δφα−δ2φϕ1μ+σμ4+α+δ+σ+ϕ1+ϕ2μ3+σ+ϕ1+ϕ2α+δ+σ+ϕ2ϕ1+σ+ϕ2δ+ϕ2σμ2+σ+ϕ2ϕ1+ϕ2σα+σ+ϕ2δ+ϕ2σϕ1+δσϕ2μ+δσϕ1ϕ2β,Vb∗=−α−φμ2+α−2φα+δμ−α2φ+−βpi−2δφα−δ2φϕ2ϕ1μ4+α+δ+σ+ϕ1+ϕ2μ3+σ+ϕ1+ϕ2α+δ+σ+ϕ2ϕ1+δ+σϕ2+δσμ2+σ+ϕ2ϕ1+ϕ2σα+δ+σϕ2+δσϕ1+δσϕ2μ+δσϕ1ϕ2β

### 3.2 The basic reproduction number

The basic reproduction number *R*_0_ of the proposed *SIV*_1_*V*_2_*V*_*b*_*S* COVID-19 model is given by:

(4) $$R_{0}=\frac{\beta \pi \left(\phi_{2}+\mu \right)\left(\phi_{1}+\mu \right)\left(\sigma +\mu \right)}{\left(\mu^{4}+\left(\sigma +\phi_{1}+\phi_{2}\right)\mu^{3}+\left(\left(\sigma +\phi_{2}\right)\phi_{1}+\sigma \phi_{2}\right)\mu^{2}+\mu \sigma \phi_{1}\phi_{2}-\varphi \phi_{1}\phi_{2}\sigma \right)\left(\alpha +\delta +\mu \right)}$$

depends on the *μ*(*ϕ*_1_ + *μ*)(*ϕ*_2_ + *μ*)(*σ* + *μ*) ≥ *σφϕ*_1_*ϕ*_2_. The *Next Generation Matrix* as seen in (Van den Driessche & Watmough, 2002) is used to obtain the basic reproduction number *R*_0_ using the system of equations in (1) which comprises two parts: *F* and *V*^−1^, where, the matrix F is the new infections, while the component of matrix V are transfers of infections from one compartment to another. $\xi_{1}^{*}$ is the COVID-19-free equilibrium state. *R*_0_ is the dominant eigenvalue of the matrix *G* = *FV*^−1^.

Thus,

(5) $$F=\left(\begin{array}{l} 0\\ \beta SI\\ 0\\ 0\\ 0 \end{array}\right),V=\left(\begin{array}{l} -\pi +\sigma V_{b}+\beta SI+\mu S\\ -\left(\alpha +\delta +\mu \right)I\\ -\varphi S-\alpha I+\left(\phi_{1}+\mu \right)V_{1}\\ -\phi_{1}V_{1}+\left(\phi_{2}+\mu \right)V_{2}\\ -\phi_{2}V_{2}+\left(\delta +\mu \right)V_{b} \end{array}\right)$$

We now obtain the *F* and *V* by computing the Jacobian F and V. Thus,

(6) $$F=\left(\begin{array}{lllll} 0 & 0 & 0 & 0\\ 0 & \beta S^{*} & 0 & 0 & 0\\ 0 & 0 & 0 & 0 & 0\\ 0 & 0 & 0 & 0 & 0\\ 0 & 0 & 0 & 0 & 0 \end{array}\right)V=\left(\begin{array}{lllll} \mu & \beta S^{*} & 0 & 0 & \sigma \\ 0 & \alpha +\delta +\mu & 0 & 0 & 0\\ -\varphi & -\alpha & \phi_{1}+\mu & 0 & 0\\ 0 & 0 & -\phi_{1} & \phi_{2}+\mu & 0\\ 0 & 0 & 0 & -\phi_{2} & \sigma +\mu \end{array}\right)$$

Now,

(7) $$FV^{-1}=\left[\begin{array}{lllll} 0 & 0 & 0 & 0 & 0\\ 0 & \frac{\beta S^{*}}{\alpha +\delta +\mu } & 0 & 0 & 0\\ 0 & 0 & 0 & 0 & 0\\ 0 & 0 & 0 & 0 & 0\\ 0 & 0 & 0 & 0 & 0 \end{array}\right]$$

Hence, the basic reproduction number *R*_0_ which is the spectra radius of *FV*^−1^ is

$$R_{0}=\rho \left(FV^{-1}\right)=\frac{\beta \pi \left(\phi_{2}+\mu \right)\left(\phi_{1}+\mu \right)\left(\sigma +\mu \right)}{\left(\mu^{4}+\left(\sigma +\phi_{1}+\phi_{2}\right)\mu^{3}+\left(\left(\sigma +\phi_{2}\right)\phi_{1}+\sigma \phi_{2}\right)\mu^{2}+\mu \sigma \phi_{1}\phi_{2}-\varphi \phi_{1}\phi_{2}\sigma \right)\left(\alpha +\delta +\mu \right)}$$

### 3.3 Equilibrium stability of equilibrium points

Consider the equilibrium points $\xi_{i}^{*}\left(S^{*},I^{*},V_{1}^{*},V_{2}^{*},V_{b}^{*}\right)$ for *i* = 1, 2. Introduce small perturbations around the equilibrium:

(8) $$\begin{array}{ll} S\left(t\right) & =S^{*}+s\left(t\right),\\ I\left(t\right) & =I^{*}+i\left(t\right),\\ V_{1}\left(t\right) & =V_{1}^{*}+v_{1}\left(t\right),\\ V_{2}\left(t\right) & =V_{2}^{*}+v_{2}\left(t\right),\\ V_{b}\left(t\right) & =V_{b}^{*}+v_{b}\left(t\right). \end{array}$$

Substituting (8) into (1) and linearising by neglecting higher order perturbation, we have.

(9) $$\frac{dy\left(t\right)}{dt}=Ay\left(t\right)+By\left(t-\tau \right),$$

where $y\left(t\right)={\left(\begin{array}{lllll} s\left(t\right) & i\left(t\right) & v_{1}\left(t\right) & v_{2}\left(t\right) & v_{b}\left(t\right) \end{array}\right)}^{T}$, and the matrices *A* and *B* are defined as follows:

Matrix *A*:

$$A=\left(\begin{array}{lllll} -\beta I^{*}-\mu & -\beta S^{*} & 0 & 0 & 0\\ \beta I^{*} & \beta S^{*}-\left(\alpha +\delta +\mu \right) & 0 & 0 & 0\\ \varphi & \alpha & -\left(\phi_{1}+\mu \right) & 0 & 0\\ 0 & 0 & \phi_{1} & -\left(\phi_{2}+\mu \right) & 0\\ 0 & 0 & 0 & \phi_{2} & -\mu \end{array}\right),B=\left(\begin{array}{lllll} 0 & 0 & 0 & 0 & \sigma \\ 0 & 0 & 0 & 0 & 0\\ 0 & 0 & 0 & 0 & 0\\ 0 & 0 & 0 & 0 & 0\\ 0 & 0 & 0 & 0 & -\sigma \end{array}\right)$$

Assuming a solution of the form **y**(*t*) = **y**_0_*e*^*λt*^ and substituting into the linearized system (9) we obtain: *λ***y**_0_*e*^*λt*^ = *A***y**_0_*e*^*λt*^ + *B***y**_0_*e*^*λ*(*t*−*τ*)^. This leads to the characteristic equation:

(10) $$\det\left(A-\lambda I+Be^{-\lambda \tau }\right)=0.$$

Proposition 3.0.1 *The COVID-*19*-free equilibrium point*$\xi_{1}^{*}=\left(S^{*},I^{*},V_{1}^{*},V_{2}^{*},V_{b}^{*}\right)$*is asymptotically stable ifR*_0_ < 1 *for* *τ* = 0.

*Proof*. The characteristic equation of (10) at the virus free state, i.e at *I*∗ = 0

(11) $$\begin{array}{ll} \lambda^{5} & -\left(-l_{5}+l_{2}-l_{3}-l_{4}-l_{1}\right)\lambda^{4}-\left(l_{1}l_{2}-l_{3}l_{1}-l_{4}l_{1}-l_{5}l_{1}+l_{3}l_{2}+l_{4}l_{2}+l_{2}l_{5}-l_{4}l_{3}-l_{5}l_{3}-l_{5}l_{4}\right)\lambda^{3}\\ & -\left(l_{1}l_{2}l_{3}+l_{1}l_{2}l_{4}+l_{1}l_{2}l_{5}-l_{1}l_{3}l_{4}-l_{1}l_{3}l_{5}-l_{1}l_{4}l_{5}+l_{2}l_{3}l_{4}+l_{2}l_{3}l_{5}+l_{2}l_{4}l_{5}-l_{3}l_{4}l_{5}\right)\lambda^{2}\\ & -\left(\sigma \varphi \phi_{1}\phi_{2}+l_{1}l_{2}l_{4}l_{3}+l_{1}l_{2}l_{3}l_{5}+l_{1}l_{2}l_{4}l_{5}-l_{1}l_{3}l_{4}l_{5}+l_{2}l_{3}l_{4}l_{5}\right)\lambda +\phi_{2}\varphi \phi_{1}\sigma l_{2}-l_{1}l_{2}l_{3}l_{4}l_{5}=0 \end{array}$$

where, *l*_1_ = *βI*∗ + *μ*, *l*_2_ = *βS*∗ − (*α* + *δ* + *μ*)*l*_3_ = (*ϕ*_1_ + *μ*)*l*_4_ = (*ϕ*_2_ + *μ*)*l*_5_ = (*μ* + *σ*) observe that if *l*_2_ = *βS*∗ − (*α* + *δ* + *μ*) < 0 i.e for *R*_0_ < 1, then the Descartes' Rule of Signs is satisfied which would result in having negative roots since all parameters are non-negative. Thus, system (1) of the model is asymptotically stable whenever *R*_0_ < 1.

Proposition 3.0.2 *The Endemic equilibrium point* (*EEP*) $\xi_{2}^{*}=\left(S^{*},I^{*},V_{1}^{*},V_{2}^{*},V_{b}^{*}\right)$ *is asymptotically stable if* *R*_0_ > 1, *for* *τ* = 0.

*Proof*. The characteristics equation of the linearized system (10) for at the endemic state is:

(12) $$\begin{array}{ll} \lambda^{5} & -\left(-l_{5}+l_{2}-l_{3}-l_{4}-l_{1}\right)\lambda^{4}-\left(-I^{*}S^{*}\beta^{2}+l_{1}l_{2}-l_{3}l_{1}-l_{4}l_{1}-l_{5}l_{1}+l_{3}l_{2}+l_{4}l_{2}+l_{2}l_{5}-l_{4}l_{3}-l_{5}l_{3}-l_{5}l_{4}\right)\lambda^{3}\\ & -\left(-I^{*}S^{*}\beta^{2}l_{3}-I^{*}S^{*}\beta^{2}l_{4}-I^{*}S^{*}\beta^{2}l_{5}+l_{1}l_{2}l_{3}+l_{1}l_{2}l_{4}\right)\\ & \left(+l_{1}l_{2}l_{5}-l_{1}l_{3}l_{4}-l_{1}l_{3}l_{5}-l_{1}l_{4}l_{5}+l_{2}l_{3}l_{4}+l_{2}l_{3}l_{5}+l_{2}l_{4}l_{5}-l_{3}l_{4}l_{5}\right)\lambda^{2}\\ & -\left(-I^{*}S^{*}\beta^{2}l_{3}l_{4}-I^{*}S^{*}\beta^{2}l_{3}l_{5}-I^{*}S^{*}\beta^{2}l_{4}l_{5}+\sigma \varphi \phi_{1}\phi_{2}+l_{1}l_{2}l_{4}l_{3}+l_{1}l_{2}l_{3}l_{5}+l_{1}l_{2}l_{4}l_{5}-l_{1}l_{3}l_{4}l_{5}+l_{2}l_{3}l_{4}l_{5}\right)\lambda \\ & +I^{*}S^{*}\beta^{2}l_{3}l_{4}l_{5}-\beta I^{*}\phi_{2}\alpha \phi_{1}\sigma +\phi_{2}\varphi \phi_{1}\sigma l_{2}-l_{1}l_{2}l_{3}l_{4}l_{5}=0 \end{array}$$

For the characteristic equation (12) to have all negative roots, the polynomial's coefficients must satisfy Descartes' Rule of Signs for *P*(−*λ*). That is the coefficients of *P*(−*λ*) should alternate in sign. Observe that the coefficients of the (12) are thus:

(13) $$\begin{array}{l} -1<0\\ \left(l_{5}-l_{2}+l_{3}+l_{4}+l_{1}\right)>0\\ \left(-I^{*}S^{*}\beta^{2}+l_{1}l_{2}-l_{3}l_{1}-l_{4}l_{1}-l_{5}l_{1}+l_{3}l_{2}+l_{4}l_{2}+l_{2}l_{5}-l_{4}l_{3}-l_{5}l_{3}-l_{5}l_{4}\right)<0\\ \left(I^{*}S^{*}\beta^{2}l_{3}+I^{*}S^{*}\beta^{2}l_{4}+I^{*}S^{*}\beta^{2}l_{5}-l_{1}l_{2}l_{3}-l_{1}l_{2}l_{4}-l_{1}l_{2}l_{5}+l_{1}l_{3}l_{4}+l_{1}l_{3}l_{5}+l_{1}l_{4}l_{5}-l_{2}l_{3}l_{4}-l_{2}l_{3}l_{5}-l_{2}l_{4}l_{5}+l_{3}l_{4}l_{5}\right)>0\\ \left(-I^{*}S^{*}\beta^{2}l_{3}l_{4}-I^{*}S^{*}\beta^{2}l_{3}l_{5}-I^{*}S^{*}\beta^{2}l_{4}l_{5}+\sigma \varphi \phi_{1}\phi_{2}+l_{1}l_{2}l_{4}l_{3}+l_{1}l_{2}l_{3}l_{5}+l_{1}l_{2}l_{4}l_{5}-l_{1}l_{3}l_{4}l_{5}+l_{2}l_{3}l_{4}l_{5}\right)<0\\ -I^{*}S^{*}\beta^{2}l_{3}l_{4}l_{5}+\beta I^{*}\phi_{2}\alpha \phi_{1}\sigma -\phi_{2}\varphi \phi_{1}\sigma l_{2}+l_{1}l_{2}l_{3}l_{4}l_{5}>0 \end{array}$$

Now, since *S*∗ > 0, *I*∗ > 0 and all the parameters of the model are positive, then for *l*_2_ = *βS*∗ − (*α* + *δ* + *μ*) > 0 i.e for *R*_0_ > 1, suppose the alternating Descartes' rule of signs in equation (13) hold, then the result follows.

### 3.4 Existence of Hopf bifurcation

The matrix for the characteristic equation when *τ* > 0, where *λ* = *iω*(*ω* > 0) is given as:

(14) $$\begin{array}{ll} & A-i\omega I+Be^{-i\omega \tau }\\ & \;=\left(\begin{array}{lllll} -\beta I^{*}-\mu -i\omega & -\beta S^{*} & 0 & 0 & \sigma e^{-i\omega \tau }\\ \beta I^{*} & \beta S^{*}-\left(\alpha +\delta +\mu \right)-i\omega & 0 & 0 & 0\\ \varphi & \alpha & -\left(\phi_{1}+\mu \right)-i\omega & 0 & 0\\ 0 & 0 & \phi_{1} & -\left(\phi_{2}+\mu \right)-i\omega & 0\\ 0 & 0 & 0 & \phi_{2} & -\mu -i\omega -\sigma e^{-i\omega \tau } \end{array}\right) \end{array}$$

To determine if a Hopf bifurcation occurs, we solve the determinant equation:

(15) $$\det\left(A-i\omega I+Be^{-i\omega \tau }\right)=0$$

At the equilibrium point *ξ*_1_, the characteristics equation is:

(16) $$\begin{array}{ll} \left(\lambda -l_{2}\right) & \left[\sigma \left(-i\omega^{3}+\left(-l_{4}-l_{1}-l_{3}\right){\left(i\omega \right)}^{2}+\left(\left(-l_{3}-l_{4}\right)l_{1}-l_{4}l_{3}\right)\left(i\omega \right)+\phi_{1}\phi_{2}\varphi -l_{1}l_{3}l_{4}\right)e^{-i\omega \tau }\right)\\ & \left(-\left(i\omega +l_{5}\right)\left(i\omega +l_{4}\right)\left(i\omega +l_{3}\right)\left(i\omega +l_{1}\right)\right]=0 \end{array}$$

that is,

(17) $$\begin{array}{ll} \sigma & \left(-i\omega_{0}^{3}-\left(-l_{4}-l_{1}-l_{3}\right)\omega_{0}^{2}+i\left(\left(-l_{3}-l_{4}\right)\right)l_{1}-l_{4}l_{3}\right)\omega_{0}+\phi_{1}\phi_{2}\varphi \left(-l_{1}l_{3}l_{4}\right)\left(\cos\left(\omega_{0}\tau \right)-\sin\left(\omega_{0}\tau \right)\right)\\ & -\left(\omega_{0}^{4}-i\left(l_{1}+l_{3}+l_{4}+l_{5}\right)\omega_{0}^{3}+\left(l_{1}l_{3}+l_{1}l_{4}+l_{3}l_{4}+l_{1}l_{5}+l_{3}l_{5}+l_{4}l_{5}\right)\omega_{0}^{2}\right)\\ & \left(+i\left(l_{1}l_{3}l_{4}+l_{1}l_{3}l_{5}+l_{1}l_{4}l_{5}+l_{3}l_{4}l_{5}\right)\omega_{0}+l_{1}l_{3}l_{4}l_{5}\right)=0. \end{array}$$

Separating the real and imaginary part we have:

(18) $$\begin{array}{ll} Re\left[f\left(\omega_{0}\right)\right] & =\sigma \left(\left(-\left(-l_{4}-l_{1}-l_{3}\right)\omega_{0}^{2}+\phi_{1}\phi_{2}\varphi -l_{1}l_{3}l_{4}\right)\right)\cos\left(\omega_{0}\tau \right)+\left(-\omega_{0}^{3}+\left(\left(-l_{3}-l_{4}\right)l_{1}-l_{4}l_{3}\right)\omega_{0}\right)\sin\left(\left(\omega_{0}\tau \right)\right)\\ & -\left(\omega_{0}^{4}-\left(l_{1}l_{3}+l_{1}l_{4}+l_{3}l_{4}+l_{1}l_{5}+l_{3}l_{5}+l_{4}l_{5}\right)\omega_{0}^{2}+l_{1}l_{3}l_{4}l_{5}\right) \end{array}$$

(19) $$\begin{array}{ll} Im\left[f\left(\omega_{0}\right)\right] & =\sigma \left(\left(-\omega_{0}^{3}+\left(\left(-l_{3}-l_{4}\right)l_{1}-l_{4}l_{3}\right)\omega_{0}\right)\cos\left(\omega_{0}\tau \right)+\left(\left(-l_{4}-l_{1}-l_{3}\right)\omega_{0}^{2}-\phi_{1}\phi_{2}\varphi +l_{1}l_{3}l_{4}\right)\right)\sin\left(\left(\omega_{0}\tau \right)\right)\\ & -\left(\left(l_{1}+l_{3}+l_{4}+l_{5}\right)\omega_{0}^{3}+\left(l_{1}l_{3}l_{4}+l_{1}l_{3}l_{5}+l_{1}l_{4}l_{5}+l_{3}l_{4}l_{5}\right)\omega_{0}\right) \end{array}$$

Taking the square of the real and thee imaginary we obtain

(20) $$\begin{array}{ll} \sigma^{2} & \left({\left(-\left(-l_{4}-l_{1}-l_{3}\right)\omega_{0}^{2}+\phi_{1}\phi_{2}\varphi -l_{1}l_{3}l_{4}\right)}^{2}+{\left(-\omega_{0}^{3}+\left(\left(-l_{3}-l_{4}\right)l_{1}-l_{4}l_{3}\right)\omega_{0}\right)}^{2}\right)\\ & ={\left(\omega_{0}^{4}-\left(l_{1}l_{3}+l_{1}l_{4}+l_{3}l_{4}+l_{1}l_{5}+l_{3}l_{5}+l_{4}l_{5}\right)\omega_{0}^{2}+l_{1}l_{3}l_{4}l_{5}\right)}^{2}+{\left(\left(l_{1}+l_{3}+l_{4}+l_{5}\right)\omega_{0}^{3}+\left(l_{1}l_{3}l_{4}+l_{1}l_{3}l_{5}+l_{1}l_{4}l_{5}+l_{3}l_{4}l_{5}\right)\omega_{0}\right)}^{2} \end{array}$$

Then we have,

(21) $$\begin{array}{ll} - & \omega_{0}^{8}+\left(\sigma^{2}+2l_{1}l_{3}+2l_{1}l_{4}+2l_{1}l_{5}+2l_{4}l_{3}+2l_{3}l_{5}+2l_{4}l_{5}-{\left(l_{1}+l_{3}+l_{4}+l_{5}\right)}^{2}\right)\omega_{0}^{6}\\ & +\left(\sigma^{2}\left({\left(l_{1}+l_{3}+l_{4}\right)}^{2}-2\left(-l_{3}-l_{4}\right)l_{1}+2l_{4}l_{3}\right)-2l_{1}l_{3}l_{4}l_{5}\right)\\ & \left(-{\left(-l_{1}l_{3}-l_{1}l_{4}-l_{1}l_{5}-l_{4}l_{3}-l_{3}l_{5}-l_{4}l_{5}\right)}^{2}-2\left(l_{1}l_{3}l_{4}+l_{1}l_{3}l_{5}+l_{1}l_{4}l_{5}+l_{3}l_{4}l_{5}\right)\left(l_{1}+l_{3}+l_{4}+l_{5}\right)\right)\omega_{0}^{4}\\ & +\left(\sigma^{2}\left(2\left(\phi_{1}\phi_{2}\varphi -l_{1}l_{3}l_{4}\right)\left(l_{1}+l_{3}+l_{4}\right)+{\left(\left(-l_{3}-l_{4}\right)l_{1}-l_{4}l_{3}\right)}^{2}\right)\right)\\ & \left(-2l_{1}l_{3}l_{4}l_{5}\left(-l_{1}l_{3}-l_{1}l_{4}-l_{1}l_{5}-l_{4}l_{3}-l_{3}l_{5}-l_{4}l_{5}\right)-{\left(l_{1}l_{3}l_{4}+l_{1}l_{3}l_{5}+l_{1}l_{4}l_{5}+l_{3}l_{4}l_{5}\right)}^{2}\right)\omega_{0}^{2}\\ & +\sigma^{2}{\left(\phi_{1}\phi_{2}\varphi -l_{1}l_{3}l_{4}\right)}^{2}-l_{1}^{2}l_{3}^{2}l_{4}^{2}l_{5}^{2}=0 \end{array}$$

Denote $p_{0}=\omega_{0}^{2}$, then Equation (21) becomes

(22) $$-p_{0}^{4}+D_{3}p_{0}^{3}+D_{2}p_{0}^{2}+D_{1}p_{0}+D_{0}=0$$

where,

$$D_{3}=\left(\sigma^{2}+2l_{1}l_{3}+2l_{1}l_{4}+2l_{1}l_{5}+2l_{4}l_{3}+2l_{3}l_{5}+2l_{4}l_{5}-{\left(l_{1}+l_{3}+l_{4}+l_{5}\right)}^{2}\right)$$

$$D_{2}=\left(\sigma^{2}\left({\left(l_{1}+l_{3}+l_{4}\right)}^{2}-2\left(-l_{3}-l_{4}\right)l_{1}+2l_{4}l_{3}\right)-2l_{1}l_{3}l_{4}l_{5}-{\left(-l_{1}l_{3}-l_{1}l_{4}-l_{1}l_{5}-l_{4}l_{3}-l_{3}l_{5}-l_{4}l_{5}\right)}^{2}-2\left(l_{1}l_{3}l_{4}+l_{1}l_{3}l_{5}+l_{1}l_{4}l_{5}+l_{3}l_{4}l_{5}\right)\left(l_{1}+l_{3}+l_{4}+l_{5}\right)\right)$$

$$D_{1}=\left(\sigma^{2}\left(2\left(\phi_{1}\phi_{2}\varphi -l_{1}l_{3}l_{4}\right)\left(l_{1}+l_{3}+l_{4}\right)+{\left(\left(-l_{3}-l_{4}\right)l_{1}-l_{4}l_{3}\right)}^{2}\right)-2l_{1}l_{3}l_{4}l_{5}\left(-l_{1}l_{3}-l_{1}l_{4}-l_{1}l_{5}-l_{4}l_{3}-l_{3}l_{5}-l_{4}l_{5}\right)-{\left(l_{1}l_{3}l_{4}+l_{1}l_{3}l_{5}+l_{1}l_{4}l_{5}+l_{3}l_{4}l_{5}\right)}^{2}\right)$$

$$D_{0}=\sigma^{2}{\left(\phi_{1}\phi_{2}\varphi -l_{1}l_{3}l_{4}\right)}^{2}-l_{1}^{2}l_{3}^{2}l_{4}^{2}l_{5}^{2}$$

Now, assuming that Equation (22) has positive roots *p*_0,1_⋯*p*_0,4_, then consequently, Equation (21) also has positive roots $\omega_{0,i}=\sqrt{p_{0,i}}$ for *i* = 1, …, 4. Thus, for *ω*_0,*i*_,

(23) $$\tau_{i}^{m}=\frac{1}{\omega_{0,i}}\cos^{-1}\left(\frac{\left(-a\omega_{0,i}^{2}+c\right)\left(\omega_{0,i}^{4}-V\omega_{0,i}^{2}+y\right)+\left(-\omega_{0,i}^{3}+b\omega_{0,i}\right)\left(U\omega_{0,i}^{3}+x\omega_{0,i}\right)}{\sigma \left({\left(-a\omega_{0,i}^{2}+c\right)}^{2}+{\left(-\omega_{0,i}^{2}+b\omega_{0,i}\right)}^{2}\right)}\right)+\frac{2m}{\omega_{0,i}},$$

*i* = 0, 1, 2, 3, ⋯, where, *a* = (−(−*l*_4_ − *l*_1_ − *l*_3_), *b* = (−*l*_3_ − *l*_4_)*l*_1_ − *l*_4_*l*_3_), *c* = *ϕ*_1_*ϕ*_2_*φ* − *l*_1_*l*_3_*l*_4_, *U* = *l*_1_ + *l*_3_ + *l*_4_ + *l*_5_, *V* = *l*_1_*l*_3_ + *l*_1_*l*_4_ + *l*_3_*l*_4_ + *l*_1_*l*_5_ + *l*_3_*l*_5_ + *l*_4_*l*_5_, *x* = *l*_1_*l*_3_*l*_4_ + *l*_1_*l*_3_*l*_5_ + *l*_1_*l*_4_*l*_5_ + *l*_3_*l*_4_*l*_5_, *y* = *l*_1_*l*_3_*l*_4_*l*_5_

Let $\tau_{0}=min\left\{\tau_{i}^{0}\right\}$, *i* = 1, 2, …, 4, then whenever *τ* = *τ*_0_, the characteristics equation (16) has roots ± *ıω*_0_ and from Equation (16) one gets:

(24) $${\left[\frac{d\lambda }{d\tau }\right]}^{-1}=-\frac{3\lambda^{2}+2q_{2}\lambda +q_{1}}{\lambda \left(\lambda^{3}+q_{2}\lambda^{2}+q_{1}\lambda +q_{0}\right)}+\frac{4\lambda^{3}+3r_{4}\lambda^{2}+2r_{3}\lambda +r_{1}}{\lambda \left(\lambda^{4}+r_{3}\lambda^{3}+r_{2}\lambda^{2}+r_{1}\lambda +r_{0}\right)}-\frac{\tau }{\lambda }$$

where, $q_{2}=\left(-l_{4}-l_{1}-l_{3}\right),q_{1}=\left(-l_{3}-l_{4}\right)l_{1}-l_{4}l_{3},q_{0}=\phi_{1}\phi_{2}\varphi -l_{1}l_{3}l_{4},r_{3}=\left(l_{5}+l_{4}+l_{3}+l_{1}\right),r_{2}=l_{4}l_{5}+\left(l_{5}+l_{4}\right)l_{3}+\left(l_{5}+l_{4}+l_{3}\right)l_{1},r_{1}=\left(l_{3}l_{4}l_{5}+\left(l_{4}l_{5}+\left(l_{5}+l_{4}\right)l_{3}\right)l_{1}\right),l_{1}l_{3}l_{4}l_{5}$. Then, for the transversality condition we have

(25) $$Re{\left[\frac{d\lambda }{d\tau }\right]}_{\lambda =i\omega_{0},\tau =\tau_{0}^{m}}^{-1}=\frac{f^{\prime }\left(p_{0}\right)}{\omega_{0}^{8}-2\omega_{0}^{6}r_{2}+\left(r_{2}^{2}+2r_{0}\right)\omega_{0}^{4}+r_{3}\omega_{0}^{3}-2\omega_{0}^{2}r_{0}r_{2}+r_{1}\omega_{0}+r_{0}^{2}}$$

where, *p*_0_ and $f\left(p_{0}\right)=-p_{0}^{4}+D_{3}p_{0}^{3}+D_{2}p_{0}^{2}+D_{1}p_{0}+D_{0}=0$. Now, if *f*′(*ρ*_0_) ≠ 0, then $Re{\left[\frac{d\lambda }{d\tau }\right]}_{\lambda =i\omega_{0},\tau =\tau_{0}^{m}}^{-1}\neq 0$. Thus, system (1) undergoes a Hopf bifurcation at the equilibrium $\xi_{1}^{*}$ when $\tau =\tau_{0}^{m},\;m=0,1,2,3,\cdots \;$.

At the equilibrium point *ξ*_2_, the characteristics equation is:

(26) $$-\left(\lambda^{4}+\left(-l_{2}+l_{3}+l_{4}+l_{1}\right)\lambda^{3}+\left(\left(-l_{2}+l_{4}+l_{1}\right)l_{3}+\left(-l_{2}+l_{1}\right)l_{4}+I^{*}S^{*}\beta^{2}-l_{1}l_{2}\right)\lambda^{2}+\left(\left(\left(-l_{2}+l_{1}\right)l_{4}+I^{*}S^{*}\beta^{2}-l_{1}l_{2}\right)l_{3}+\left(I^{*}S^{*}\beta^{2}-l_{1}l_{2}\right)l_{4}-\phi_{1}\phi_{2}\varphi \right)\lambda +\left(I^{*}S^{*}\beta^{2}-l_{1}l_{2}\right)l_{4}l_{3}-\phi_{2}\phi_{1}\left(I^{*}\alpha \beta -\varphi l_{2}\right)\right)\sigma e^{-\lambda \tau }-\left(\lambda^{5}+\left(l_{3}-l_{2}+l_{1}+l_{5}+l_{4}\right)\lambda^{4}+\left(l_{3}\left(-l_{2}+l_{1}\right)+I^{*}S^{*}\beta^{2}-l_{1}l_{2}+\left(l_{3}-l_{2}+l_{1}\right)l_{5}+\left(l_{3}-l_{2}+l_{1}+l_{5}\right)l_{4}\right)\lambda^{3}+\left(l_{3}\left(I^{*}S^{*}\beta^{2}-l_{1}l_{2}\right)+\left(l_{3}\left(-l_{2}+l_{1}\right)+I^{*}S^{*}\beta^{2}-l_{1}l_{2}\right)l_{5}+\left(l_{3}\left(-l_{2}+l_{1}\right)+I^{*}S^{*}\beta^{2}-l_{1}l_{2}+\left(l_{3}-l_{2}+l_{1}\right)l_{5}\right)l_{4}\right)\lambda^{2}+\left(l_{3}\left(I^{*}S^{*}\beta^{2}-l_{1}l_{2}\right)l_{5}+\left(l_{3}\left(I^{*}S^{*}\beta^{2}-l_{1}l_{2}\right)+\left(l_{3}\left(-l_{2}+l_{1}\right)+I^{*}S^{*}\beta^{2}-l_{1}l_{2}\right)l_{5}\right)l_{4}\right)\lambda +l_{3}\left(I^{*}S^{*}\beta^{2}-l_{1}l_{2}\right)l_{5}l_{4}\right)=0.$$

let.

$a_{3}=\left(-l_{2}+l_{3}+l_{4}+l_{1}\right)$
$a_{2}=\left(\left(-l_{2}+l_{4}+l_{1}\right)l_{3}+\left(-l_{2}+l_{1}\right)l_{4}+I^{*}S^{*}\beta^{2}-l_{1}l_{2}\right)$
$a_{1}=\left(\left(\left(-l_{2}+l_{1}\right)l_{4}+I^{*}S^{*}\beta^{2}-l_{1}l_{2}\right)l_{3}+\left(I^{*}S^{*}\beta^{2}-l_{1}l_{2}\right)l_{4}-\phi_{1}\phi_{2}\varphi \right)$
$a_{0}=\left(I^{*}S^{*}\beta^{2}-l_{1}l_{2}\right)l_{4}l_{3}-\phi_{2}\phi_{1}\left(I^{*}\alpha \beta -\varphi l_{2}\right)$
$b_{4}=\left(l_{3}-l_{2}+l_{1}+l_{5}+l_{4}\right)$
$b_{3}=\left(l_{3}\left(-l_{2}+l_{1}\right)+I^{*}S^{*}\beta^{2}-l_{1}l_{2}+\left(l_{3}-l_{2}+l_{1}\right)l_{5}+\left(l_{3}-l_{2}+l_{1}+l_{5}\right)l_{4}\right)$
$b_{2}=\left(l_{3}\left(I^{*}S^{*}\beta^{2}-l_{1}l_{2}\right)+\left(l_{3}\left(-l_{2}+l_{1}\right)+I^{*}S^{*}\beta^{2}-l_{1}l_{2}\right)l_{5}+\left(l_{3}\left(-l_{2}+l_{1}\right)+I^{*}S^{*}\beta^{2}-l_{1}l_{2}+\left(l_{3}-l_{2}+l_{1}\right)l_{5}\right)l_{4}\right)$
$b_{1}=\left(l_{3}\left(I^{*}S^{*}\beta^{2}-l_{1}l_{2}\right)l_{5}+\left(l_{3}\left(I^{*}S^{*}\beta^{2}-l_{1}l_{2}\right)+\left(l_{3}\left(-l_{2}+l_{1}\right)+I^{*}S^{*}\beta^{2}-l_{1}l_{2}\right)l_{5}\right)l_{4}\right)$
$b_{0}=l_{3}\left(I^{*}S^{*}\beta^{2}-l_{1}l_{2}\right)l_{5}l_{4}$

Separating the real and imaginary part of the characteristics equation (26) at *λ* = *iω*(*ω* > 0), where *e*^−*iωτ*^ = cos(*ωτ*) − *i* sin(*ωτ*) we have:

(27) $$\left\{\begin{array}{l} Re\left[f\left(\omega \right)\right]=\sigma \left(-\omega^{4}+a_{2}\omega^{2}-a_{0}\right)\cos\left(\omega \tau \right)+\sigma \left(a_{3}\omega^{3}-a_{1}\omega \right)\sin\left(\omega \tau \right)-\left(b_{4}\omega^{4}-b_{2}\omega^{2}+b_{0}\right)\;\\ Im\left[f\left(\omega \right)\right]=\sigma \left(\omega^{4}-a_{2}\omega^{2}+a_{0}\right)\sin\left(\omega \tau \right)+\sigma \left(a_{3}\omega^{3}-a_{1}\omega \right)\cos\left(\omega \tau \right)-\left(\omega^{5}-b_{3}\omega^{3}+b_{1}\omega \right)\; \end{array}\right)$$

Taking the square of the real and imaginary part and adding we have

(28) $$\sigma^{2}\left({\left(-\omega^{4}+a_{2}\omega^{2}-a_{0}\right)}^{2}+{\left(a_{3}\omega^{3}-a_{1}\omega \right)}^{2}\right)={\left(b_{4}\omega^{4}-b_{2}\omega^{2}+b_{0}\right)}^{2}+{\left(\omega^{5}-b_{3}\omega^{3}+b_{1}\omega \right)}^{2}$$

then we obtain

(29) $$\omega^{10}+\left(-\sigma^{2}+b_{4}^{2}-2b_{3}\right)\omega^{8}+\left(-\sigma^{2}\left(a_{3}^{2}-2a_{2}\right)+b_{3}^{2}+2b_{1}-2b_{2}b_{4}\right)\omega^{6}+\left(-\sigma^{2}\left(-2a_{1}a_{3}+a_{2}^{2}+2a_{0}\right)-2b_{1}b_{3}+2b_{0}b_{4}+b_{2}^{2}\right)\omega^{4}+\left(-\sigma^{2}\left(-2a_{0}a_{2}+a_{1}^{2}\right)+b_{1}^{2}-2b_{0}b_{2}\right)\omega^{2}-\sigma^{2}a_{0}^{2}+b_{0}^{2}=0$$

Denote *ρ* = *ω*^2^, then Equation (29) becomes

(30) $$\rho^{5}+d_{4}\rho^{4}+d_{3}\rho^{3}+d_{2}\rho^{2}+d_{1}\rho +d_{0}=0$$

where.

$d_{4}=\left(-\sigma^{2}+b_{4}^{2}-2b_{3}\right)$
$d_{3}=\left(-\sigma^{2}\left(a_{3}^{2}-2a_{2}\right)+b_{3}^{2}+2b_{1}-2b_{2}b_{4}\right)$
$d_{2}=\left(-\sigma^{2}\left(-2a_{1}a_{3}+a_{2}^{2}+2a_{0}\right)-2b_{1}b_{3}+2b_{0}b_{4}+b_{2}^{2}\right)$
$d_{1}=\left(-\sigma^{2}\left(-2a_{0}a_{2}+a_{1}^{2}\right)+b_{1}^{2}-2b_{0}b_{2}\right)$
$d_{0}=-\sigma^{2}a_{0}^{2}+b_{0}^{2}$

Now, assuming that Equation (30) has positive roots *ρ*_1_⋯*ρ*_5_, then consequently, Equation (29) also has positive roots $\omega_{i}=\sqrt{\rho_{i}}$ for *i* = 1, ⋯5. For *ω*_*i*_,

(31) $$\tau_{i}^{m}=\frac{i}{\omega }\cos^{-1}\left[\frac{\left(-\omega^{4}+a_{2}\omega^{2}-a_{0}\right)\left(b_{4}\omega^{4}-b_{2}\omega^{2}+b_{0}\right)+\left(a_{3}\omega^{3}-a_{1}\omega \right)\left(\omega^{5}-b_{3}\omega^{3}+b_{1}\omega \right)}{\sigma {\left(-\omega^{4}+a_{2}\omega^{2}-a_{0}\right)}^{2}+{\left(a_{3}\omega^{3}-a_{1}\omega \right)}^{2}}\right]+\frac{2m\pi }{\omega_{i}},\;i=0,1,2,3,\cdots$$

Let $\tau_{0}=min\left\{\tau_{i}^{0}\right\},\;i=1,2,3,\ldots ,5$, then whenever *τ* = *τ*_0_, the characteristics equation (17) has roots ± *iω*_0_, and from (17) one gets:

(32) $${\left[\frac{d\lambda }{d\tau }\right]}^{-1}=\frac{4\lambda^{3}+3a_{3}\lambda^{2}+2a_{2}\lambda +a_{1}}{\lambda \left(\lambda^{4}+a_{3}\lambda^{3}+a_{2}\lambda^{2}+a_{1}\lambda +a_{0}\right)}-\frac{5\lambda^{4}+4b_{4}\lambda^{3}+3b_{3}\lambda^{2}+2b_{2}\lambda +b_{1}}{\lambda \left(\lambda^{5}+b_{4}\lambda^{4}+b_{3}\lambda^{3}+b_{2}\lambda^{2}+b_{1}\lambda +b_{0}\right)}-\frac{\tau }{\lambda }$$

Then, for the transversality condition we have

(33) $$Re{\left[\frac{d\lambda }{d\tau }\right]}_{\lambda =i\omega_{0},\tau =\tau_{0}^{m}}^{-1}=\frac{f^{\prime }\left(\rho_{0}\right)}{\omega_{0}^{10}+\left(b_{4}^{2}-2b_{3}\right)\omega_{0}^{8}+\left(-2b_{2}b_{4}+b_{3}^{2}+2b_{1}\right)\omega_{0}^{6}+\left(2b_{0}b_{4}-2b_{1}b_{3}+b_{2}^{2}\right)\omega_{0}^{4}+\left(-2b_{0}b_{2}+b_{1}^{2}\right)\omega_{0}^{2}+b_{0}^{2}}$$

where, *ρ*_0_ and $f\left(\rho_{0}\right)=\rho_{0}^{5}+d_{4}\rho_{0}^{4}+d_{3}\rho_{0}^{3}+d_{2}\rho_{0}^{2}+d_{1}\rho_{0}+d_{0}$. Now, if *f*′(*ρ*_0_) ≠ 0, then $Re{\left[\frac{d\lambda }{d\tau }\right]}_{\lambda =i\omega_{0},\tau =\tau_{0}^{m}}^{-1}\neq 0$. Thus, system (1) undergoes a Hopf bifurcation at the equilibrium $\xi_{2}^{*}$ when $\tau =\tau_{0}^{m},\;m=0,1,2,3,\cdots \;$. Hence, in the light of the existence of Hopf bifurcation and coupled with the Hopf bifurcation theorem in (Yang, Wang, Kundu, Song, & Zhang, 2022), we conclude with the following Theorem 3.1.

Theorem 3.1 *IfR*_0_ > 1, *then the endemic equilibrium point* $\xi_{2}^{*}$ *is locally asymptotically stable for* *τ* ∈ (0, *τ*_0_); *the system also undergoes bifurcation at the endemic point* $\xi_{2}^{*}$ *as* *τ* *approaches near t* *τ*_0_.

### 3.5 Global stability analysis of DFE and EEP

#### 3.5.1 Global stability of the COVID-19-free equilibrium pont (DFE)

First, we shall show the condition for the global stability of the system when there are no COVID-19 infectious persons in the population.

Theorem 3.2 *The COVID-*19 *free equilibrium point of model (1) is globally asymptotically stable forR*_0_ < 1

*Proof*. Consider the following Lyapunov function

(34) $$U_{1}\left(S^{*},I^{*},V_{1}^{*},V_{2}^{*},V_{b}^{*}\right)=w_{1}S^{*}+w_{2}I^{*}+w_{3}V_{1}^{*}+w_{4}V_{2}^{*}+w_{5}V_{b}^{*}$$

then,

(35) $$U_{1}^{\prime }=w_{1}\left(\pi +\sigma V_{b}^{*}\left(t-\tau \right)-\beta S^{*}I^{*}-\mu S^{*}\right)+w_{2}\left(\beta S^{*}I^{*}-\left(\alpha +\delta +\mu \right)I^{*}\right)+w_{3}\left(\varphi S^{*}+\alpha I^{*}-\left(\phi_{1}+\mu \right)V_{1}^{*}\right)+w_{4}\left(\phi_{1}V_{1}^{*}-\left(\phi_{2}+\mu \right)V_{2}^{*}\right)+w_{5}\left(\phi_{2}V_{2}^{*}-\sigma V_{b}^{*}\left(t-\tau \right)-\mu V_{b}^{*}\right)$$

Now, taking *w*_1_ = *w*_3_ = *w*_4_ = *w*_5_ = 0, one obtains,

$$\begin{array}{lll} U_{1}^{\prime } & = & w_{2}\left(\beta S^{*}I^{*}-\left(\alpha +\delta +\mu \right)I^{*}\right)\\ & = & w_{2}\left(\frac{\beta S^{*}I^{*}}{\alpha +\delta +\mu }-I^{*}\right)\\ & = & w_{2}\left(R_{0}-1\right)I^{*}<0,if\;R_{0}<1 \end{array}$$

Showing that this is Lyapunov function whenever *R*_0_ < 1. Also, $U_{1}^{\prime }=0$ if only *I* = 0. Implying that the trajectory of the solution for which $U_{1}^{\prime }=0$ is the COVID-19-free equilibrium point $\xi_{1}^{*}$. Thus, the largest compact invariant set $\left\{\left(S,I,V_{1},V_{2},V_{b}\right)\in R_{+}^{5}:U_{1}^{\prime }=0\right\}$ is the singleton set $\left\{\xi_{1}^{*}\right\}$, is the invariant set. Hence, by the LaSalle's invariant principle, the COVID-19 free equilibrium point (DFE) is globally asymptotically stable in $R_{+}^{5}$ if *R*_0_ < 1. This completes the proof.

#### 3.5.2 Global stability of the endemic equilibrium point (EEP)

Theorem 3.3 *The COVID-*19 *endemic point given by*$\xi_{2}^{*}=\left(S^{*},I^{*},V_{1}^{*},V_{2}^{*},V_{b}^{*}\right)$*is asymptotically stable in*$R_{+}^{5}$*ifR*_0_ > 1.

*Proof*. Consider a Lyapunov function whose domain Ω, defined by:

(36) $$U_{2}\left(S,I,V_{1},V_{2},V_{b}\right)=\left(S-S^{*}-S^{*}log\frac{S}{S^{*}}\right)+\left(I-I^{*}-I^{*}log\frac{I}{I^{*}}\right)+\left(V_{1}-V_{1}^{*}-V_{1}^{*}log\frac{V_{1}}{V_{1}^{*}}\right)+\left(V_{2}-V_{2}^{*}-V_{2}^{*}log\frac{V_{2}}{V_{2}^{*}}\right)+\left(V_{b}-V_{b}^{*}-V_{b}^{*}log\frac{V_{b}}{V_{b}^{*}}\right)$$

Differentiating *U*_2_(*S*, *I*, *V*_1_, *V*_2_, *V*_*b*_) along the trajectory of the solution of the model (.) we obtain:

(37) $$\begin{array}{ll} \frac{dU_{2}}{dt} & =\left(\frac{S-S^{*}}{S}\right)\frac{dS}{dt}+\left(\frac{I-I^{*}}{I}\right)\frac{dI}{dt}+\left(\frac{V_{1}-V_{1}^{*}}{V_{1}}\right)\frac{dV_{1}}{dt}+\left(\frac{V_{2}-V_{2}^{*}}{V_{2}}\right)\frac{dV_{2}}{dt}+\left(\frac{V_{b}-V_{b}^{*}}{V_{b}}\right)\frac{dV_{b}}{dt}\\ & =\left(\frac{S-S^{*}}{S}\right)\left(\pi +\sigma V_{b}\left(t-\tau \right)-\beta SI-\mu S\right)\\ & +\left(\frac{I-I^{*}}{I}\right)\left(\beta SI-\left(\alpha +\delta +\mu \right)I\right)+\left(\frac{V_{1}-V_{1}^{*}}{V_{1}}\right)\left(\varphi S+\alpha I-\left(\left(\phi_{1}+\mu \right)V_{1}\right)\right)\\ & +\left(\frac{V_{2}-V_{2}^{*}}{V_{2}}\right)\left(\phi_{1}V_{1}-\left(\phi_{2}+\mu \right)V_{2}\right)\\ & +\left(\frac{V_{b}-V_{b}^{*}}{V_{b}}\right)\left(\phi_{2}V_{2}-\left(\sigma \left(t-\tau \right)+\mu \right)V_{b}\right) \end{array}$$

(38) $$\begin{array}{ll} \frac{dU_{2}}{dt} & =\left(\frac{S-S^{*}}{S}\right)\left(\pi +\sigma V_{b}\left(t-\tau \right)-\beta I\left(S-S^{*}\right)-\mu \left(S-S^{*}\right)+\beta IS^{*}+\mu S^{*}\right)\\ & +\left(\frac{I-I^{*}}{I}\right)\left(\beta S\left(I-I^{*}\right)-\left(\alpha +\delta +\mu \right)\left(I-I^{*}\right)+\beta SI^{*}+\left(\alpha +\delta +\mu \right)I^{*}\right)\\ & +\left(\frac{V_{1}-V_{1}^{*}}{V_{1}}\right)\left(\varphi S+\alpha I-\left(\phi_{1}+\mu \right)\left(V_{1}-V_{1}^{*}\right)+\left(\left(\phi_{1}+\mu \right)V_{1}^{*}\right)\right)\\ & +\left(\frac{V_{2}-V_{2}^{*}}{V_{2}}\right)\left(\phi_{1}V_{1}-\left(\phi_{2}+\mu \right)\left(V_{2}-V_{2}^{*}\right)+\left(\phi_{2}+\mu \right)V_{2}^{*}\right)\\ & +\left(\frac{V_{b}-V_{b}^{*}}{V_{b}}\right)\left(\phi_{2}V_{2}-\left(\sigma \left(t-\tau \right)+\mu \right)\left(V_{b}-V_{b}^{*}\right)+\left(\sigma \left(t-\tau \right)+\mu \right)V_{b}^{*}\right) \end{array}$$

Observe that we can write $\frac{dU_{2}}{dt}$ as *ℓ*_1_ − *ℓ*_2_, where

(39) $$\begin{array}{ll} \ell_{1} & =\pi +\sigma V_{b}\left(t-\tau \right)+\left(\beta I+\mu \right)S^{*}+\frac{{\left(I-I^{*}\right)}^{2}}{I}\left(\beta S-\left(\alpha +\delta +\mu \right)\right)\\ & +\left(\beta S-\left(\alpha +\delta +\mu \right)\right)I^{*}+\varphi S+\mu I+\left(\phi_{1}+\mu \right)V_{1}^{*}+\phi_{1}V_{1}+\left(\phi_{2}+\mu \right)V_{2}^{*}\phi_{2}V_{2}+\left(\sigma \left(t-\tau \right)\right)V_{b}^{*} \end{array}$$

(40) $$\ell_{2}=\frac{S^{*}}{S}\left(\pi +\sigma V_{b}\left(t-\tau \right)\right)+\frac{{\left(S-S^{*}\right)}^{2}}{S}\left(\beta I+\mu \right)+\frac{S^{*2}}{S}\left(\beta I+\mu \right)+\frac{I^{*2}}{I}\left(\beta S+\left(\alpha +\delta +\mu \right)\right)+\frac{V_{1}^{*}}{V_{1}}\left(\varphi S+\alpha I\right)+\frac{{\left(V_{1}-V_{1}^{*}\right)}^{2}}{V_{1}}\left(\phi_{1}+\mu \right)+\frac{V_{1}^{*2}}{V_{1}}\left(\phi_{1}+\mu \right)+\frac{V_{2}^{*}}{V_{2}}\phi_{1}V_{1}+\frac{{\left(V_{2}-V_{2}^{*}\right)}^{2}}{V_{2}}\left(\phi_{2}+\mu \right)+\frac{V_{2}^{*2}}{V_{2}}\left(\phi_{2}+\mu \right)+\frac{V_{b}^{*}}{V_{b}}\phi_{2}V_{2}+\frac{{\left(V_{b}-V_{b}^{*}\right)}^{2}}{V_{b}}\left(\sigma \left(t-\tau \right)+\mu \right)$$

Showing that when *βS*∗ − (*α* + *δ* + *μ*) > 0 that is for the endemic point $\frac{\beta S^{*}}{\alpha +\delta +mu}>1$, that is *R*_0_ > 1 then $\frac{dU_{2}}{dt}\leq 0$ for *ℓ*_1_ ≤ *ℓ*_2_ and $\frac{dU_{2}}{dt}=0$ if and only if *ℓ*_1_ = *ℓ*_2_ (this is true since all parameters in the model are non-negative). Observe immediately that $\frac{dU_{2}}{dt}=0$ if and only if *S* = *S*∗, $V_{1}=V_{1}^{*}$, $V_{2}=V_{2}^{*}$, $V_{b}=V_{b}^{*}$. Hence, by LaSalle's invariant principle, the result follows.

## 4 Numerical simulations, fittings and parameter estimation

To solve and simulate the results of the proposed model, we used the ‘dde23‘ solver in MATLAB, which is an efficient and accurate method for solving delay differential equations by integrating the Runge-Kutta method. To ensure accurate simulations, we first fit our model to real data to estimate the parameters instead of assuming random values. The final parameter values obtained from the numerical simulations for *I*, *V*_1_, *V*_2_, and *V*_*b*_ are 100.4486, 6.3615, 5.4410, and 133.3400, respectively, as shown in Table 3.

**Table 3:** Summary of estimated parameter.

Parameter	First estimation	Second estimation	Numerical solution
*π*	0.9999	1.0000	1.0000
*σ*	0.9997	0.9898	0.09898
*β*	0.0057	0262	0.0262
*μ*	0.0000	0.0355	0.0355
*α*	0.0374	0.0330	0.0330
*δ*	0.0426	0.9835	0.14835
*φ*	0.9989	0.9835	09835
*ϕ*_1_	1.0000	0.9979	0.6979
*ϕ*_2_	1.0000	0.9897	0.9879
*τ*	–	–	150

The fitting process utilized one-year COVID-19 data from May 1, 2023 to April 30, 2024, sourced from (Ministry of Health (MOH), 2023). For parameter estimation, we employed the ‘lsqcurvefit‘ function in MATLAB, which uses nonlinear least squares optimization to minimize the difference between the observed and predicted data. The optimization was constrained using the ‘fmincon‘ function to avoid overfitting. To further mitigate overfitting, cross-validation techniques were used by dividing the dataset into training and testing subsets, and regularization was applied to constrain the parameter space (see Fig. 2).

**Fig. 3:**
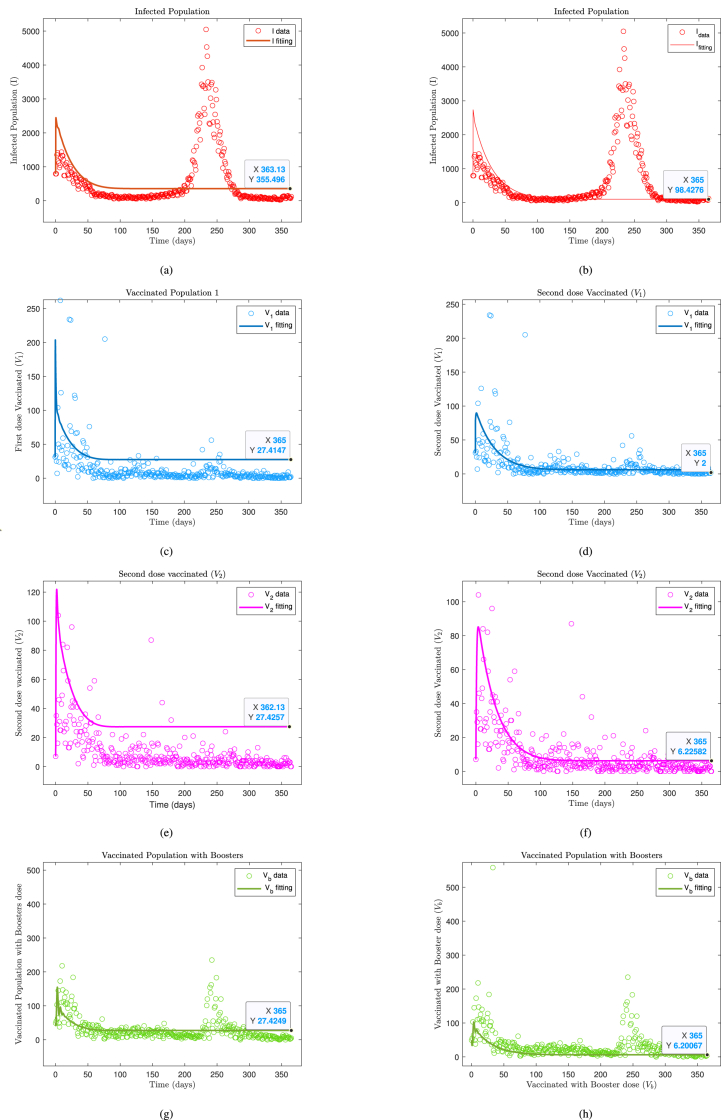
Fittings of the *SIV*_1_*V*_2_*V*_*b*_*S* model with the Malaysia daily report data from May 1, 2023 to April 30, 2024.

For the numerical simulation, projecting for the next 24 months, the final value for the infected class (*I*) drops to 11 per day, while the vaccinated groups stabilize with a booster dose (*V*_*b*_) increasing to 106 per day. This shows the long-term dynamics of the COVID-19 spread with respect to the vaccination strategy, and the results are illustrated in Fig. 3.

### 4.1 Numerical simulation of the Hopf bifurcation

**Fig. 4:**
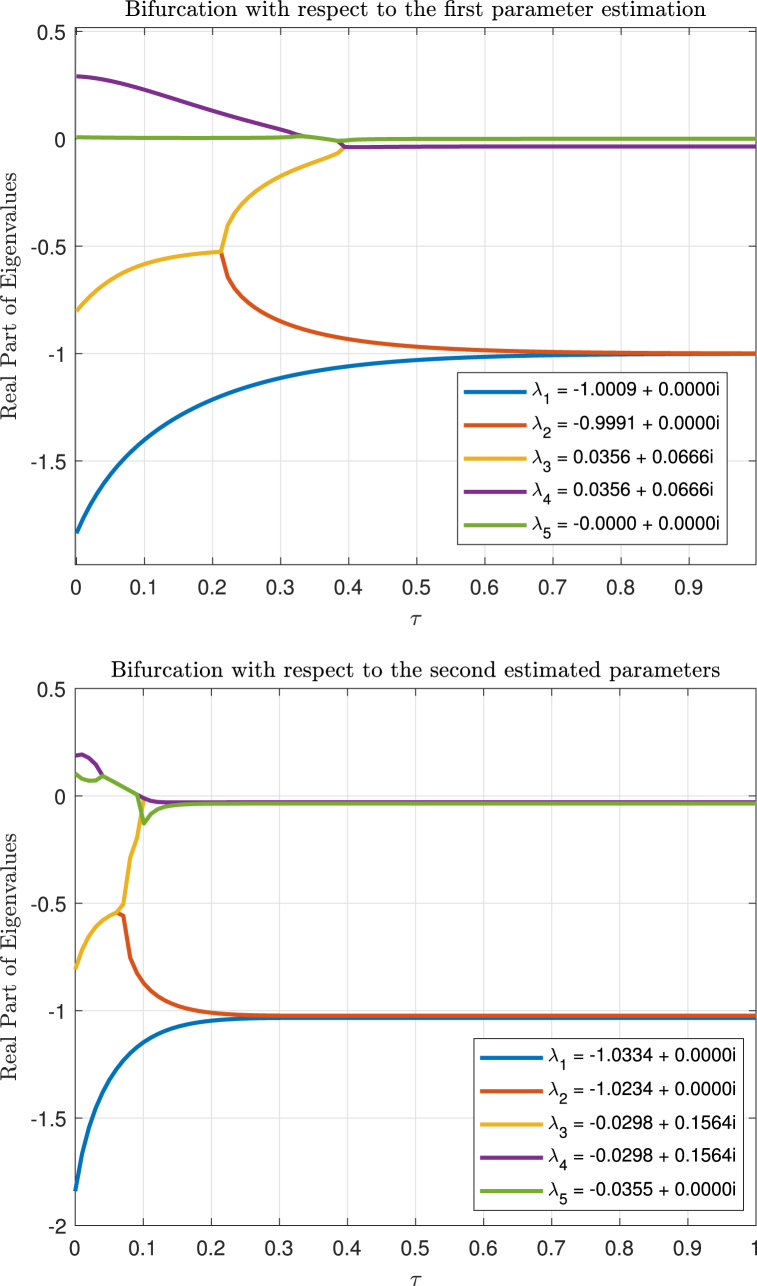
Bifurcations with respect to estimated parameters.

The Hopf bifurcation simulation was carried out using the ‘dde23‘ solver, as it is well-suited for delay differential systems. The bifurcation occurs at *τ* = 0.89899 with complex conjugate eigenvalues close to the imaginary axis, signaling the onset of oscillatory behavior in the system. The eigenvalues and bifurcation points are shown in Equation (41), and the corresponding figure for bifurcation is provided in Fig. 4. This confirms the presence of a Hopf bifurcation under the estimated parameters.

(41) $$\begin{array}{l} \lambda_{1}=-1.0334+0.0000i,\\ \lambda_{2}=-1.0234+0.0000i,\\ \lambda_{3}=-0.0298+0.1564i,\\ \lambda_{4}=-0.0298-0.1564i,\\ \lambda_{5}=-0.0355+0.0000i \end{array}$$

#### 4.1.1 The herd immunity threshold (HIT)

The Herd immunity, also known as population immunity, ”is the indirect protection from an infectious disease that happens when a population is immune either through vaccination or immunity developed through previous infection” (WHO, 2019). The threshold for herd immunity is *HIT* = 1 − (1/*R*_0_) (Fine, Eames, & Heymann, 2011). Now, taking the waning period for the booster dose be 5 months (≈150days) (Menni et al., 2022), and the largest value of the real part of the eigenvalues obtained in (40) that is −1.0334, we obtain *R*_0_ = 0.70155. Thus, $HIT=1-\frac{1}{0.70155}=1-1.42542=-0.42542$, which is −0.43%. ”Herd immunity typically ranges from 0% to 100%. However, in our case, it falls significantly below this range. This is because our basic reproduction number (*R*_0_) is less than 1, indicating stability. On average, each infected individual is transmitting the virus to less than one other person. This suggests that the number of new COVID-19 cases will naturally decline, leading to the eventual disappearance or fading away of the virus as the number of new cases decreases over time.”

## 5 Uncertainty and sensitivity analysis

Uncertainty analysis (UA) is all about finding and measuring possible errors connected to an estimated parameter. The aim is to measure the impact of a parameter based on different inputs. Meanwhile, sensitivity analysis (SA) helps understand how a parameter affects a dependent variable given certain assumptions. It's vital grasping a model's behavior.

For capturing the uncertainty of estimated parameters, confidence intervals were computed for each one. This data was then used to determine the confidence interval for the basic reproduction number. To conduct sensitivity analysis, the Latin Hypercube Sampling with partial rank correlation coefficient index (LHS-PRCC) method was utilized, focusing on assessing each parameter's influence on the basic reproduction number as per Marino (2008).

**Fig. 5:**
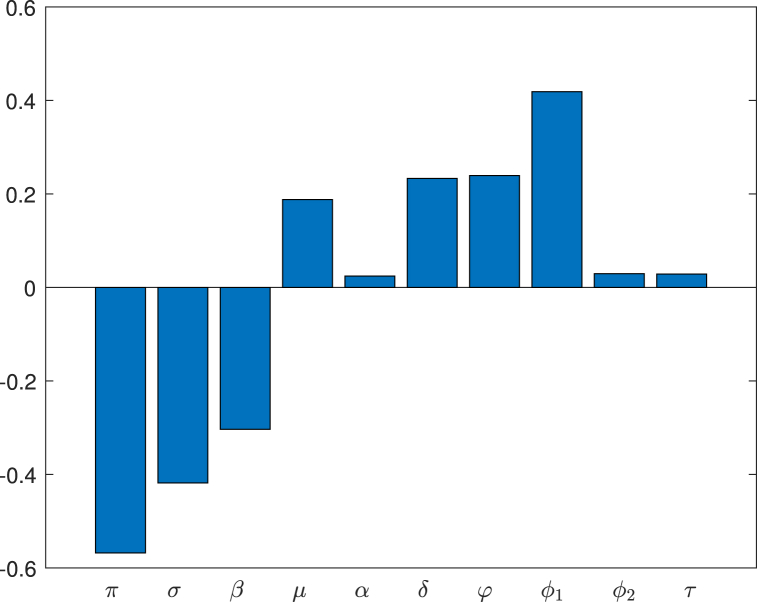
The PRCC of the influence of each parameter on the basic reproduction number (*R*_0_).

The sensitivity indices of the basic reproduction number in the *SIVS* model were acquired using the LHS-PRCC method with 5000 samples of each parameter as inputs. In Fig. 5, you can see a visual representation of the PRCC, with bar size denoting the parameter's impact on disease dynamics. A slight change in a highly influential parameter could result in significant shifts in disease dynamics.

**Fig. 6:**
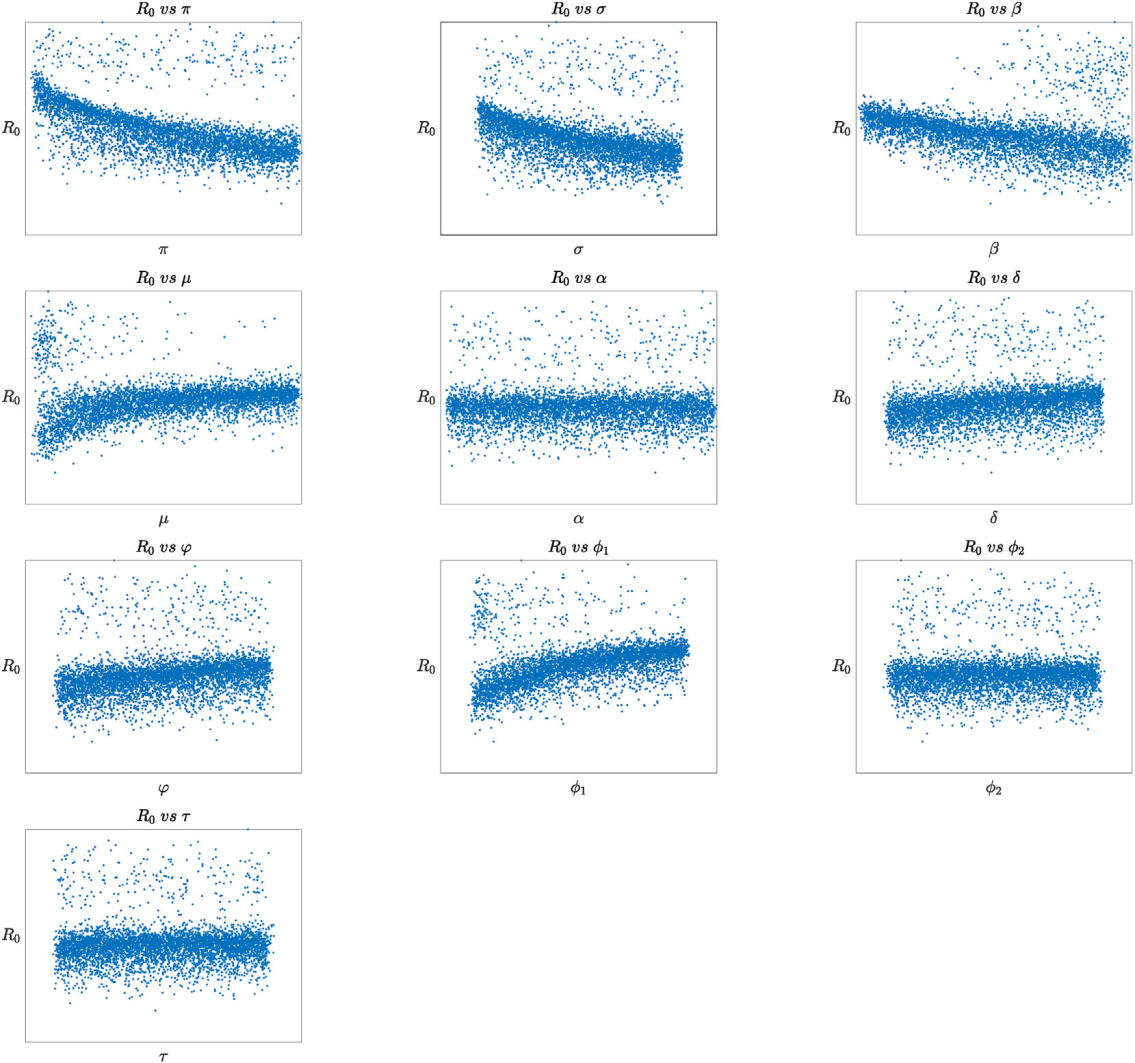
Scatter plots showing the relationship of the model parameters on the basic reproduction number *R*_0_.

Positive indices indicate a direct relationship with the reproduction number – boosting these parameters increases its value. Conversely, negative indices signal an inverse relationship – raising these values decreases the reproduction number. For a detailed breakdown of sensitivity indices, refer to Table 4. Furthermore, Fig. 6 illustrates each parameter's connection with the basic reproduction number *R*_0_.

**Table 4:** Summary of Parameter and Sensitivity indices.

Parameter	Sensitivity indices	Parameter	Sensitivity indices
*π*	−0.5680	*σ*	−0.4182
*β*	−0.3033	*μ*	0.1879
*α*	0.0243	*δ*	0.0232
*φ*	0.2393	*ϕ*_1_	0.4187
*ϕ*_2_	0.0295	*τ*	0.6286

## 6 Results and discussion

The analytical results first confirm that the model system has a non-negative solution, implying that all the compartments in the model remain biologically feasible. The stability analysis of the COVID-19-free equilibrium reveals that it is locally asymptotically stable when the basic reproduction number *R*_0_ is less than 1, indicating that the infection will eventually die out in the absence of external interventions, as shown in previous models using the Next Generation Matrix (Van den Driessche & Watmough, 2002). When *R*_0_ is greater than 1, the endemic equilibrium becomes stable, and a time delay (*τ*) contributes to the model's dynamic behavior, including the possibility of bifurcations.

The results indicate that the fitted value of *R*_0_ is 0.70155, much lower than values estimated earlier in the pandemic. This decrease is attributed to widespread vaccination and other control measures, such as social distancing and mask-wearing, which have been shown to significantly reduce transmission rates. The reduction of *R*_0_ to below 1 indicates that each infected individual is infecting fewer than one other person, suggesting that the spread of the virus is effectively controlled and will likely decline.

The model also estimates the herd immunity threshold, though lower than conventional thresholds, due to the reduced basic reproduction number. This suggests that, at this point, the combination of natural immunity, vaccination, and control measures has a significant impact on limiting the spread of the virus, and further vaccinations will help maintain this control.

The model predicts a significant decline in the number of infected individuals (*I*) over time, with daily cases dropping to around 11 within the next two years. This trend can be biologically interpreted as the result of successful vaccination campaigns that boost immunity in the population. The increasing numbers of individuals receiving booster doses (*V*_*b*_) support this, as booster doses have been shown to enhance immunity, especially in those with weakened or waning immune responses. The initial decline in first-dose (*V*_1_) and second-dose (*V*_2_) vaccinations reflects the progression of individuals moving through the stages of full vaccination to receiving booster doses.

Biologically, the waning of immunity is accounted for in the model, showing that despite the rise in booster doses, there remains a small but stable number of individuals returning to the susceptible class due to the waning of immunity after vaccination. This reflects real-world dynamics where periodic boosters are needed to maintain immunity against variants of the virus.

The forecasting period for future dynamics of COVID-19 has been explicitly set to 24 months, as shown in the results of our numerical simulations. Over this period, we project that the number of daily infected individuals will decrease significantly, while booster vaccinations will continue to play a crucial role in sustaining herd immunity.

## 7 Summary and conclusion

In response to the need for understanding the post-COVID-19 vaccination dynamics in a heterogeneous population, with Malaysia as a case study, we developed an extended SIVS epidemiological model. This model uniquely incorporates the waning immunity period following booster doses, reflecting the fact that COVID-19 vaccines do not provide permanent immunity. The model was solved using MATLAB's Runge-Kutta method for delay differential equations, ensuring an accurate representation of the system dynamics.

One year of daily data (from May 1, 2023 to April 30, 2024) was used for fitting and parameter estimation. The estimated basic reproduction number was calculated as less than one, confirming the system's stability and reflecting the declining trend in new COVID-19 cases. Furthermore, the bifurcation analysis of the model showed all negative eigenvalues, confirming that the system is stable around the estimated parameters.

A comparison of the final values from the numerical simulations with the actual data shows a strong correlation, validating the accuracy of the model. For example, the final values for infected individuals, first-dose, second-dose, and booster-dose vaccinations were approximately 100, 6, 5, and 133, respectively, closely matching the observed data. Projections over the next two years forecast a significant reduction in the number of infected individuals and an increase in booster-dose vaccinations, with future values estimated at 11, 1, 1, and 106, respectively.

The novelty of this work lies in its incorporation of waning immunity, use of recent real-life data for model validation, and comprehensive sensitivity analysis. These quantitative insights provide a valuable tool for public health management, particularly in regions with similar vaccination and infection patterns. The model's predictive capability offers reliable support for decision-making in ongoing efforts to control the COVID-19 pandemic.

## CRediT authorship contribution statement

**Emmanuel A. Nwaibeh:** Writing – original draft, Methodology, Conceptualization. **Majid K.M. Ali:** Supervision.

## Declaration of competing interest

The authors of this research entitled “Religion a Major Predictor of Preventive Behaviours During the COVID-19 Pandemic: A structural Model” hereby declare that we have no competing or conflicting interests either personal or financially that has any influence on this work.
